# Extensive mass spectrometry proteomics data of *Persicaria minor* herb upon methyl jasmonate treatment

**DOI:** 10.1016/j.dib.2017.09.063

**Published:** 2018-02-08

**Authors:** Wan Mohd Aizat, Sarah Ibrahim, Reyhaneh Rahnamaie-Tajadod, Kok-Keong Loke, Hoe-Han Goh, Normah Mohd Noor

**Affiliations:** Institute of Systems Biology (INBIOSIS), Universiti Kebangsaan Malaysia, 43600 UKM Bangi, Selangor, Malaysia

## Abstract

Proteomics is often hindered by the lack of protein sequence database particularly for non-model species such as *Persicaria minor* herbs. An integrative approach called proteomics informed by transcriptomics is possible [1], in which translated transcriptome sequence database is used as the protein sequence database. In this current study, the proteome profile were profiled using SWATH-MS technology complemented with documented transcriptome profiling [2], the first such report in this tropical herb. The plant was also elicited using a phytohormone, methyl jasmonate (MeJA) and protein changes were elucidated using label-free quantification of SWATH-MS to understand the role of such signal molecule in this herbal species. The mass spectrometry proteomics data was deposited to the ProteomeXchange Consortium via the PRIDE partner repository with the dataset identifier PXD005749. This data article refers to the article entitled “Proteomics (SWATH-MS)-informed by transcriptomics approach of *Persicaria minor* leaves upon methyl jasmonate elicitation” [3].

**Specifications Table** *[please fill in right-hand column of the table below]*

**Table:** 

Subject area	*Plant biology*
More specific subject area	*Proteomics and plant science*
Type of data	*Figures, raw data*
How data was acquired	*Samples were analyzed using nanoLC-Ultra 2Dplus system (Eksigent Technologies, Dublin, CA, USA) coupled with TripleTOF 5600 mass spectrometer (AB SCIEX Foster City, CA, USA)*
Data format	*Processed, analyzed*
Experimental factors	*Non-treated control and methyl jasmonate (MeJA) treated Persicaria minor herbal leaf*
Experimental features	*P. minor herbal proteome description and relative quantification. The total proteins were reduced, alkylated and tryptic digested before desalted and subjected to LC-MS/MS run using both information-dependent acquisition (IDA) experiments and SWATH-MS.*
Data source location	*UKM Bangi, Malaysia (2°55′09.0″N 101°47′04.8″E)*
Data accessibility	*Data are available via the PRIDE partner repository with the dataset identifier*PXD005749

**Value of the data**

• The data described the most comprehensive proteome study in *Persicaria minor* herb.
• Integrative and complementary approach of proteomics informed by transcriptomics successfully identify various complex mixture of proteins in this non-model organism.
• SWATH-MS is a new technological advance with high-throughput analysis and allows label-free quantification.
• This dataset can be used as a protein catalogue to identify potential biomarkers for MeJA hormonal response.

## 1 Data

**Fig. S1:**
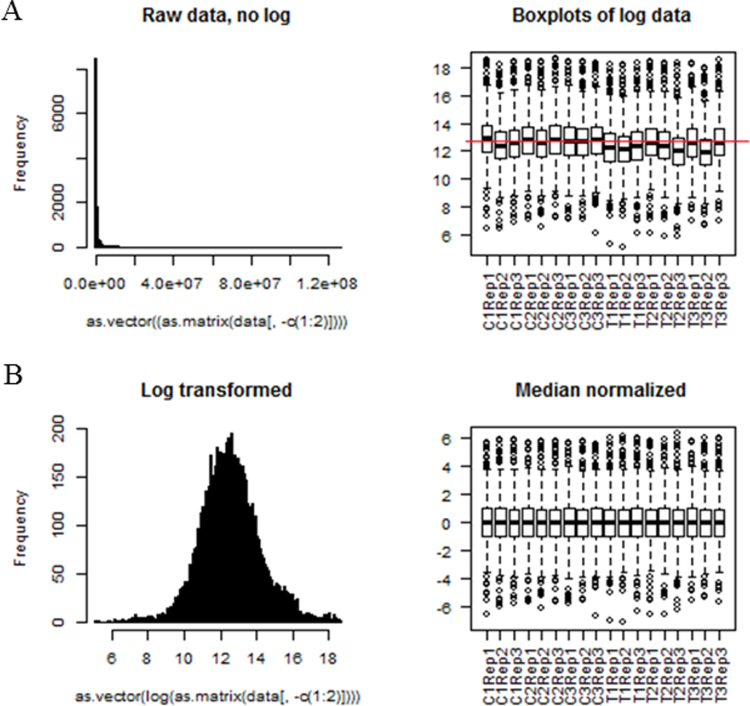
The data distribution and boxplot of identified protein peak areas from *Persicaria minor* herb (A). The areas were then log-transformed (since otherwise the data distribution is very long-tailed) and median normalized (B).

**Fig. S2:**
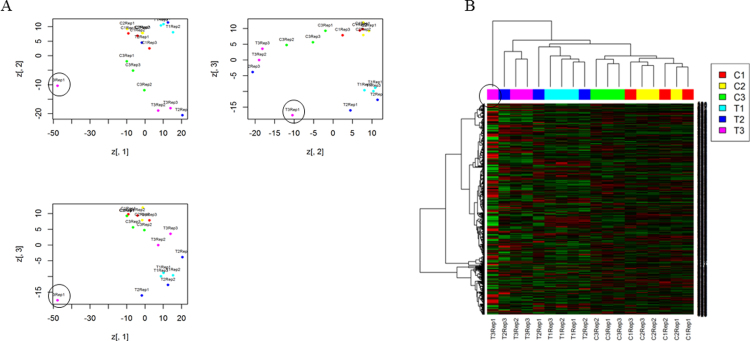
The normalized peak areas data for the identified proteins of *Persicaria minor* were further examined using principal component analysis (PCA) (A) and clustering (B). Three biological replicates for both control (C1, C2 and C3) and methyl jasmonate treated (T1, T2 and T3) samples as well as three technical replicates (rep 1, rep 2 and rep 3) for each biological replicate were run using SWATH-MS analysis. The technical replicate T3 rep 1 (circled) was then removed from subsequent analyses due to being an outlier.

A comprehensive protein profile is presented from the proteome analysis of *P. minor* herb treated with methyl jasmonate (MeJA). Both information-dependent acquisition (IDA) as well as SWATH-MS analyses were performed to generate spectrum library as well as to identify and quantify proteins, respectively. All the raw data have been deposited in the PRIDE database with the identifier of PXD005749. The data distribution (Fig. S1) and initial multivariate analyses (Fig. S2) were also reported. This available information would be valuable to further comprehend the proteins involved in MeJA elicitation and how such hormone affects their differential regulation.

## 2 Experimental design, materials and methods

Both *P. minor* untreated control (C) and MeJA treated (24 h after treatment) samples (T) were prepared as detailed in [2]. Total proteins were extracted and analyzed from three biological and three technical replicates from each control or treated group as described in [3]. Briefly, 100 mg grounded samples were mixed in TRI-reagent (1 mL) as well as chloroform (200 µL) and centrifuged (2000×*g* for 5 min). Resulting pellets were washed once using absolute ethanol, three times with absolute acetone, another three times with 1 mL guanidine hydrochloride (in 95% ethanol and 2.5% glycerol) and lastly once with 2.5% glycerol in absolute ethanol. Resulting pellets were air-dried before sent to Australian Proteome Analysis Facility (APAF), Sydney for proteomics analysis.

### 2.1 Sample preparation

Protein pellets were thoroughly mixed in 1% sodium deoxycholate in 100 mM triethyl ammonium bicarbonate (TEAB). Samples were heated at 95°C for 5 min, reduced with dithiothreitol (DTT), alkylated with iodoacetamide (IAA) and tryptic digestion was performed overnight at 37°C. Peptides were desalted using solid phase extraction with OMIX C18 and dried before solubilized in 40 µL 2% acetonitrile and 0.1% formic acid.

### 2.2 Proteome analysis

Peptide samples were separated using NanoLC-Ultra 2Dplus system (Eksigent Technologies, Dublin, CA, USA) before identified using TripleTOF 5600 mass spectrometer (AB SCIEX Foster City, CA, USA) as described in [3]. Briefly, samples were first run through a trap column (Halo C18 solid core, 100 µm × 3.5 cm) before sent to a separation column (Halo C18 solid core, 100 µm × 20 cm). Peptides were separated over 80 min at a flow rate of 400 nL/min. A linear gradient was performed from 95:5 to 60:40 mobile phase A/mobile phase B (mobile phase A: 0.1% v/v formic acid; mobile phase B: 80% v/v ACN containing 0.1% v/v formic acid). Information-dependent acquisition (IDA) was conducted using a survey scan of 350 to 1500 Da followed by 20 MS/MS product ion scan. SWATH-MS was then performed using a MS scan of 350 to 1500 Da followed by 60 MS/MS product ion scan in 12.5 Da windows.

### 2.3 Protein identification and label-free quantitation

ProteinPilot version 4.2.0.0 (AB SCIEX Foster City, CA, USA) was used to generate spectral library from the IDA runs and protein identity was determined using an in-house translated transcriptome database [2], [4]. PeakView software version 2.0.0.9257 was utilized to quantitate protein level with peptide filter setting of 6 transitions per peptide, 25 peptides per protein, 99% peptide confidence and a 1% False Discovery Rate.

### 2.4 Statistical analysis

Peak areas were log-transformed and median normalized to produce a normal data distribution using PeakView (Fig. S1). Principal component analysis (PCA) and clustering were also performed (Fig. S2). One technical replicate (T3 replicate 1) was considered an outlier (circled in Fig. S2). All other statistical analyses were performed as detailed in [3].
